# The Association of Childhood Allergic Diseases with Prenatal Exposure to Pollen Grains Through At-Birth DNA Methylation

**DOI:** 10.3390/epigenomes9010009

**Published:** 2025-03-11

**Authors:** Rajesh Melaram, Hongmei Zhang, James Adefisoye, Hasan Arshad

**Affiliations:** 1 College of Nursing and Health Sciences, Texas A&M University-Corpus Christi, Corpus Christi, TX 78412, USA; rajesh.melaram@tamucc.edu; 2 Division of Epidemiology, Biostatistics and Environmental Health, School of Public Health, University of Memphis, Memphis, TN 38152, USA; james.adefisoye@memphis.edu; 3 David Hide Asthma and Allergy Research Centre, St. Mary’s Hospital, Newport PO30 5TG, UK; s.h.arshad@soton.ac.uk

**Keywords:** allergic rhinitis, asthma, epigenetics, mediation, pregnancy

## Abstract

Background: Pollen exposure in early life is shown to be associated with allergy and asthma. DNA methylation (DNAm), an epigenetic marker, potentially reacts to pollen. However, the role of at-birth DNAm between prenatal pollen grain (PPG) exposure and childhood asthma and allergic rhinitis is unknown. Methods: Data in a birth cohort study on the Isle of Wight, UK, were analyzed (*n* = 236). Newborn DNAm was measured in cord blood or blood spots on Guthrie cards and screened for potential association with PPG exposure using the R package ttScreening. CpGs that passed screening were further assessed for such associations via linear regressions with adjusting covariates included. Finally, DNAm at PPG-associated CpGs were evaluated for their association with asthma and allergic rhinitis using logistic regressions, adjusting for covariates. The impact of cell heterogeneity on the findings was assessed. Statistical significance was set at *p* < 0.05. Results: In total, 42 CpGs passed screening, with 41 remaining statistically significant after adjusting for covariates and cell types (*p* < 0.05). High PPG exposure was associated with lower DNAm at cg12318501 (*ZNF99*, β = −0.029, *p* = 0.032) and cg00929606 (*ADM2*, β = −0.023, *p* = 0.008), which subsequently was associated with decreased odds of asthma (OR = 0.11, 95% CI 0.02–0.53, *p* = 0.006; OR = 0.14, 95% CI 0.02–1.00, *p* = 0.049). For rhinitis, cg15790214 (*HCG11*) was shown to play such a role as a mediator (β = −0.027, *p* ≤ 0.0001; OR = 0.22, 95% CI 0.07–0.72, *p* = 0.01). Conclusions: The association of PPG exposure with childhood asthma and allergic rhinitis incidence is potentially mediated by DNAm at birth.

## 1. Introduction

Allergic diseases such as asthma, rhinitis, and eczema are common inflammatory diseases in childhood. Atopic diseases as such are the result of shared genetic, epigenetic, and environmental factors [1]. In 2019, the global prevalence and incidence of childhood asthma was approximately 81 million and 20 million, respectively [2]. In Europe, asthma affects 70 million people, while 100 million suffer from allergic rhinitis [3]. These diseases account for a substantial burden in healthcare costs and a diminished quality of life [4]. It is estimated that EUR 3.03 billion is spent annually on patient services for asthma across the UK [5]. Additionally, >25,000 UK children with asthma attacks are admitted to hospitals each year, contributing to an annual loss of 2.8 million school days [6].

Despite limited epidemiological data on environmental risk factors and allergic diseases, exposure to pollen grains has been shown as a risk factor for asthma and allergic diseases [7]. Heavy rain, high relative humidity, and pollutants are known to influence the release of pollen grains [8]. When humidity is high, pollen grains can rupture, causing winds to disperse allergens in the atmosphere [9]. The three main plant sources of allergic pollen grains are grasses, trees, and weeds [10]. In Europe, approximately 20% of the population is affected by grass pollen allergies [11]. Group 1 (β-expansins) and group 5 allergens constitute major grass pollen allergens of pollen-sensitized patients [12]. The group 1 allergens Phl p 1 and Phl p 5 are two well-studied markers of timothy grass (*Phleum pratense*) allergy [13]. In a prospective observational birth cohort study, Phl p 1 was identified as an initiator of IgE sensitization in >75% of children with grass pollen-related seasonal allergic rhinitis, followed by antibody production in response to seven other allergic molecules (i.e., Phl p 2, Phl p 4, Phl p 5, Phl p 6, Phl p7, Phl p 11, and Phl p 12) of *P. pratense* [14]. Group 5 allergens only appear in the Pooideae subfamily of grasses and possess ribonuclease activity [10]. Allergens from this group are known to induce severe asthma attacks in sensitized patients [13].

Highly allergic pollen-producing tree genera span throughout Europe, including *Betula* (birch), *Cupressus* (cypress), and *Olea* (olive) [15]. Patients with birch pollinosis, for example, may experience IgE-mediated allergic reactions in response to cross-reacting allergies present in fruits, nuts, roots, and vegetables [16]. For these patients, allergic reactions to rosaceous fruits can result from the pathogen-related (PR)-10 components Mar d 1 in apples and Pur p 1 in pears, which share a structural homology with the major birch pollen allergen, Bet v 1 [17,18]. The pollen allergen Cup s 7 from can cross-react with gibberellin-regulated proteins in fruits, contributing to the development of pollen–food allergy syndrome (PFAS) [19]. Clinical manifestations of PFAS include gastrointestinal upset, respiratory symptoms, and rhinitis [18]. In severe cases, patients might suffer from itchy skin, wheezing episodes, vomiting, and anaphylaxis [20].

Epidemiological studies investigating the relationship between early life exposure to pollen (i.e., daily counts, season of birth, etc.) and asthma outcomes are scant. For instance, one study suggested that an infant’s persistent exposure to daily ambient levels of pollen grains increases the risk of asthma and allergic rhinitis at age 6/7 years [21]. Two studies observed associations between pollen season of birth and risk of atopic sensitization in children [22,23]. Offspring born between February and July and who had early onset of symptoms were at an increased risk of pollen sensitization in the first 15 months of life [22]. Maternal exposure to high levels of birch pollen counts were less sensitized than children’s exposure to high levels of birch pollen counts in the first year of life [23]. On the contrary, few studies have reported that high pollen exposure during pregnancy is associated with increased risk of asthma hospitalization admissions or incident asthma [24,25]. The daily total grass pollen concentrations accumulated between 2005 and 2011 were associated with increased adult hospitalizations for asthma in London, UK [24].

DNA methylation (DNAm) is a prime epigenetic mechanism that involves the addition of methyl groups to cytosine residues at specific cytosine-phosphate-guanine (CpG) dinucleotides across the epigenome. DNAm regulates gene functions without changing the DNA sequence and plays a key role in various cellular process including cell differentiation, gene regulation, genomic imprinting, and preservation of chromosome stability [26,27]. CpGs may cluster in GC-rich regions (CpG islands) within gene-regulatory elements of the DNA sequence, including promoters or enhancers, modulating gene expression activity [7]. Only few studies to date have examined the impact of pollen grain exposure or pollen season on DNAm changes and their potential effects on allergic diseases [28,29]. Subjects exposed to rye grass pollen grains (2000–3500 grains/m^3^) for 3 h on two consecutive days in an environmental exposure units showed a significant decrease in DNAm at cg27107150 in *TPSG1* [28]. During the pollen season, patients with co-occurrence of allergic rhinitis and chronic spontaneous urticaria generally had lower DNAm levels, in which a total of 98 differentially methylation positions (72 hypomethylated and 18 hypermethylated) were detected [29]. However, it is unclear whether the impact of pollen grain exposure during the prenatal period induces DNAm changes in newborn offspring.

It has been suggested that DNAm may serve as a molecular mediator between season of birth in early life and health outcomes later in life [30]. For example, offspring born in autumn and associated with high DNAm levels and season-associated CpGs were concurrently associated with allergic outcomes, including eczema, in 18-year-old participants [31]. An epigenome-wide meta-analysis concluded that associations between season of birth and DNAm at birth or childhood are temporally unique throughout the year (i.e., seasonal) [30]. Findings from a retrospective cohort study found that autumn- or winter-born babies had a higher risk of adult asthma, where those born in any time of the time year (with summer as the reference group) had a higher risk of allergic rhinitis [32]. Compared to season of birth, the level of exposure to pollen grains during pregnancy carries more concrete information with respect to the severity of exposure. To our knowledge, no studies have examined the association between pollen grain exposure and DNAm; moreover, the mediating role of DNAm between prenatal pollen grain (PPG) exposure and allergic diseases is unclear. To this end, through a longitudinal method, we examined the association of PPG exposure with DNAm at birth and studied to what extent the PPG exposure-associated DNAm is further associated with the incidence of asthma and allergic rhinitis in early childhood. We chose a longitudinal analysis to eliminate the concern of reverse causation with a clear time order.

In this study, we aimed to examine the potential mediating role of newborn DNAm on the association of PPG exposure with childhood asthma and allergic rhinitis incidence in a longitudinal birth cohort. We hypothesized that certain CpG sites were involved in the connection between PPG exposure and childhood allergic diseases as mediators.

## 2. Results

### 2.1. Descriptive Statistics

Maternal risk factors during pregnancy were included, such as smoking, asthma or wheeze status, and rhinitis status. Socioeconomic status (SES), sex of offspring, and passive smoke exposure during the first year of life were also examined. No statistically significant differences in these factors were observed between the complete cohort and subcohort. For clinical diagnoses, the proportion of subjects with childhood asthma at age 6 in the complete cohort was 7.20% compared to 13.56% in the subcohort (*p* < 0.05). The proportions of subjects with allergic rhinitis at ages 2, 3, and 6 in the subcohorts were consistent with those in the complete cohorts, with the largest proportions of allergic rhinitis at age 6 (20.99% in the subcohort vs. 19.51% in the complete cohort, Table 1).

PPG exposure included the sum of all pollen counts over the course of full-term pregnancy (40 weeks). For study subjects with DNAm, the average level of PPG exposure was 12,489.36 grains/m^3^ compared to 10,780.29 grains/m^3^ among the complete cohort (*p* < 0.05).

### 2.2. Pollen Grain Exposure in Pregnancy Is Associated with DNA Methylation at Birth

**Figure 1:**
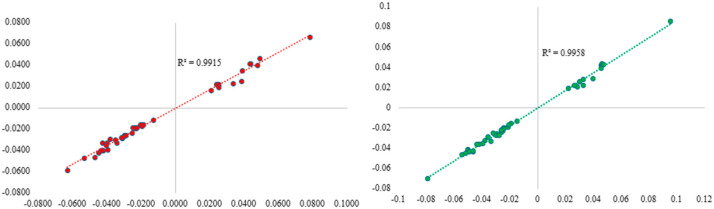
Correlation between regression coefficient estimates for 41 PPG-associated CpG sites before (x-axis) and after cell type adjustment (y-axis) in relation to asthma (**left**) and allergic rhinitis (**right**) direction. The complete results are in Table S1B,C.

The screening process identified a total of 42 CpG sites potentially associated with pollen grain exposure in pregnancy based on data in IOWTBC (Table S1A). We listed the top 10 most significant CpGs associated with PPG exposure, along with their biological relevance, from the screening process (Table 2). Since the status of maternal asthma or wheeze and that of maternal rhinitis showed a statistically significant association (χ^2^ = 13.14, *p* = 0.0003) in a complete model, to avoid collinearity, we included these two confounders in two separate linear models along with other confounders (maternal smoking, maternal asthma or wheeze, SES, and sex). With maternal asthma or wheeze included in the model (along with other confounders), 41 of the 42 CpGs remained associated with PPG exposure at significance level 0.05 (Table S1B). When maternal rhinitis was included in the model instead of maternal asthma or wheeze, all 42 CpGs remained statistically significant (Table S1C). When using cell type-adjusted DNAm at the identified CpGs and the same sets of confounders, the direction of association was consistent with those without cell type adjustment (Figure 1). In subsequent analyses (second arm), we focused on the 41 CpGs to maximize the informativity of these CpGs. Of these 41 CpGs, 16 (39%) were in the promoter region (TSS sites).

### 2.3. Relationship of Prenatal Pollen Grain-Associated CpGs with Childhood Asthma

We further examined the relationship of PPG-associated CpGs with childhood asthma at age 6 years. Of the 41 PPG-associated CpGs, DNAm at cg12318501 (located at TSS1500 of gene *ZNF99*) and cg00929606 (located at TSS1500 of gene *ADM2*) showed statistically significant associations with decreased odds of asthma, adjusting for maternal asthma or wheeze, SES, passive smoke exposure, and sex (OR = 0.23, 95% CI 0.07–0.81, *p* = 0.022; OR = 0.16, 95% CI 0.03–0.98, *p* = 0.048). The odds at CpGs cg12318501 and cg00929606 were further reduced when using cell type-adjusted DNAm (OR = 0.11, 95% CI 0.02–0.53 *p* = 0.0059; OR = 0.14, 95% CI 0.02–1.00, *p* = 0.049). The associations were not statistically significant at the remaining 39 CpGs (Table S2A).

### 2.4. Relationship of Prenatal Pollen Grain-Associated CpGs with Childhood Allergic Rhinitis

We also examined the relationship of the 41 PPG-associated CpGs with childhood allergic rhinitis at ages 2, 3, and 6 years using generalized linear models with repeated measures. After adjusting for the same confounders (except that maternal asthma was replaced with maternal rhinitis) as those for childhood asthma, PPG-associated CpG cg15790214, located in the body of gene *HCG11*, was shown to be associated with increased odds of allergic rhinitis (OR = 4.16, 95% CI 1.41–12.27, *p* = 0.0099). When using cell type-adjusted DNAm, the odds of allergic rhinitis were significantly reduced (OR = 0.22, 95% CI 0.07–0.72, *p* = 0.01). The associations were not statistically significant at the remaining 40 CpGs (Table S2B).

## 3. Discussion

We explored the role of DNAm at birth on the association of pollen grain exposure during pregnancy and the incidence of childhood allergic diseases, asthma, and allergic rhinitis. In the first arm of our study, after adjusting for confounders, we observed that high pollen grain exposure during the prenatal period was associated with at-birth DNAm at 41 CpG sites. Among the PPG-associated CpG sites, cg11676546, located at and mapped to gene *CHD7*, demonstrated epigenetic relevance. *CHD7* encodes the chromodomain helicase DNA binding protein 7, an enzymatic protein involved in chromatin remodeling to regulate gene activity [33]. However, cg11676546 did not show statistical significance in relation to asthma or allergic rhinitis.

Two PPG-associated CpGs, cg12318501 and cg00929606, were shown to be associated with decreased odds of asthma incidence at age 6 years, and such association was not impacted by cell type heterogeneity, although statistical significance disappeared when using data from Guthrie cards, which was likely due to small sample size (*n* = 44). CpGs cg12318501 and cg00929606 were located at TSS1500 of gene *ZNF99* and TSS1500 of gene *ADM2*, respectively. Research has shown that *ADM2* plays a protective role in tissue damage in cardiovascular, renal, and pulmonary systems [34]. An in vitro study found that adrenomedullin 2 is an important vasorelaxant in the pulmonary vascular system, indicating a protective effect in hypoxia-induced pulmonary hypertensive rats [35]. The *ZNF99* gene encodes a protein of the zinc finger protein family, zinc finger protein 99, and is involved in negative regulation of gene transcription. One study showed that genes encoding zinc finger proteins, including *ZNF99*, were associated with the pathogenesis of bronchial asthma in the Volga-Ural region of Russia [36]. Our findings seem to support these two CpG sites, cg12318501 and cg00929606, as being protective to the risk of asthma since we observed that a higher pollen grain exposure during pregnancy was associated with a lower level in DNAm, which was further associated with a reduced risk of asthma. It has been suggested that children with higher exposure to pollen grains tend to have a higher risk of asthma [37], but findings of these two CpG sites from our study seem to support their potential to reduce such an impact. Subsequent studies are warranted to further examine this possibility.

Our assessment on PPG-associated CpGs with allergic rhinitis showed that DNAm at the CpG site cg15790214 was associated with decreased odds of allergic rhinitis. This CpG site was located in the body of gene *HCG11*, which encodes for HLA complex group 11. *HCG11* has been linked to various health outcomes, including gastric tumors [38], glioma [39], and lung cancer [40]. A recent study found high levels of *HCG11* expressed in nasopharyngeal carcinoma tissues [41]. Based on our findings, children who have lower DNAm at PPG-associated cg15790214 are also at a decreased risk of developing allergic rhinitis. Future studies are desired to examine the potential of these three CpG sites, namely cg1218501, cg00929606, and cg15790214, with respect to their potential for allergic disease prediction, respectively, which may help in prevention of asthma and allergic rhinitis at a much earlier stage of life.

This study has several strengths. Foremost, to our knowledge, this is the first cohort study to investigate the role of DNAm at birth and the connection between pollen grain exposure in pregnancy and the development of childhood asthma and allergic rhinitis. For our study design, we used time-lagged modeling rather than mediation analyses to focus on the potential mediating role of DNAm between PPG exposure and childhood asthma incidence. As such, we were able to understand the PPG exposure on DNAm at birth and the subsequent impact of DNAm on childhood asthma and allergic rhinitis incidence. We also examined how the findings were influenced by cell type compositions. In most epigenetic studies, cell type compositions are almost always adjusted in data analyses, regardless of the need for such adjustment [42]. Our results without adjusting for cell type compositions showed relatively consistent findings on the strength and direction of associations with those when cell type compositions were adjusted. This observation indicated that cell type adjustment may not always be needed. Furthermore, the screening of CpGs allowed us to exclude CpGs that were unlikely to be informative. At the genome scale, it was agreed that informative loci tend to be sparse [43], and conducting full analyses directly at the genome scale will reduce statistical power and introduce a risk of false-positive conclusions. Hence, screening before full analyses is potentially beneficial in omics studies.

In terms of the limitations, this study included methylation sites from two distinct platforms, Illumina Infinium Human Methylation450 BeachChip and Illumina Infinium MethylationEPIC BeadChip, leading to potential bias in DNAm measurement. However, it has been shown these two platforms have strong agreement, with an overall correlation > 0.90 for all matched samples [44]. It is desirable to replicate the study in an independent cohort, preferably with comparable population features for the purpose of maximizing statistical power. We also did not analyze gene expression levels of the identified PPG-associated CpGs in connection with asthma and allergic rhinitis in early childhood. Such information can be used to validate the biological relevance of cg1218501, cg00929606, and cg15790214 in childhood allergic diseases. In our study, DNAm was measured in blood instead of airway tissues, and thus, the identified CpGs have a potential to serve as markers but not causal factors. Future studies are warranted to examine the role of airway DNAm sites in the association of aeroallergen exposures and the development of childhood allergic diseases, along with their related gene expression patterns.

## 4. Materials and Methods

### 4.1. The Isle of Wight Third Generation Birth Cohort (IOWTBC)

The Isle of Wight (IoW) third-generation birth cohort (IOWTBC) study included children born between 2010 and 2022 (*n* = 611), and at least one of their parents were in the IoW birth cohort established in 1989–1990 (*n* = 1456). The IOWTBC was followed at ages 3, 6, and 12 months and 2, 3, and 6 years [45,46]. Standardized questionnaires were used to collect information on demographics, clinical outcomes, and related risk factors. Recruitment of the IOWTBC during pregnancy was approved by the National Research Ethics Service (NRES) Committee South Central—Hampshire B (09/H0504/129) committee, along with informed consent of parental assessment and follow-up for their children (2 years—REC no. 14/SC/0133; 3 years—REC no. 14/SC/1191; and NRES Committee East Midlands—Leicester Central for 6–7 years—REC no. 17/EM/0083) [46].

### 4.2. Outcome Variables

Childhood asthma and allergic rhinitis were the main outcomes of this study and were collected based on responses to the questionnaires derived from the International Study of Asthma and Allergy in Childhood (ISAAC). Asthma was defined as a physician-diagnosed asthma or noninfectious wheeze accompanied by either inhaled steroid or bronchodilator treatment [47]. Status of asthma at age 6 years and status of allergic rhinitis at ages 2, 3, and 6 years were examined.

### 4.3. Exposure Variable

Pollen grain exposure during pregnancy is the main exposure variable in this study. During the period from 1 March 1992 to 27 August 2012, daily pollen counts (grains/m^3^) from 12 plants (*Corylus*, *Alder*, *Salix*, *Betula*, *Fraxinus*, *Ulmus*, *Quercus*, *Platanus*, *Gramineae*, *Urtica*, *Artemisia*, and *Ambrosia*) were conducted. A Burkard Volumetric Spore Trap was used to collect pollen spores on the roof of St. Mary’s Hospital, IoW, England. Pollen exposure during pregnancy was estimated as the sum of pollen counts (grains/m^3^) accumulated from a child’s presumed date of conception to their birth date, on the basis that their mother’s pregnancy lasted 280 days (40 weeks) [25].

### 4.4. DNA Methylation

DNA was isolated from cord blood (*n* = 192) or blood spots on Guthrie cards (*n* = 44) in offspring with pollen exposure. Per the manufacturer’s standard protocol, the EZ 96-DNA methylation kit (Zymo Research, Tustin, CA, USA) was used to bisulfite treat 1 µg of DNA for cytosine to thymine conversion. Subsequently, epigenome-wide DNA methylation (DNAm) was measured by the Illumina Infinium Human Methylation450 BeadChip (>484,000 CpGs) or, when available, Illumina Infinium MethylationEPIC BeadChip (>850,000 CpGs) (Illumina, Inc., San Diego, CA, USA). CpGs common between the two platforms were included in the study. Furthermore, CpG sites on sex chromosomes (X and Y) or with single nucleotide polymorphism (SNP) within 10 base pairs and with a minor allele frequency >0.007 were excluded to avoid bias [48].

Methylation levels of CpG sites were expressed as beta (*β*) values ranging from 0 (no cytosine methylation) to 1 (complete cytosine methylation), which is defined as the proportion of methylated (M) sites divided by the sum of methylated (M) and unmethylated (U) sites (*β* = M/(c + M + U)), where c is a constant to avoid dividing by 0. Because *β* values close to 0 or 1 generally suffer from heteroscedasticity, they were logit-transformed (log_2_(*β*/(1 − *β*)) to M-values [49]. Finally, batch effects were adjusted by utilizing an empirical Bayes approach implemented in the R package ComBat 1.6 [50]. After pre-processing and quality control, a total of 294,265 CpG sites were included for analyses.

### 4.5. Cell Type Composition Correction

DNAm measured in cord blood or Guthrie cards were possibly affected by heterogenous cell compositions. To address such a potential impact, cell compositions were estimated using the estimateCellCounts function in the R package minfi 1.53.1. A reference panel from Bakulski et al. was used to estimate the cell type proportions in cord blood [51], and the cells included B cells, CD4 + T cells, CD8 + T cells, granulocytes, monocytes, natural killer (NK) cells, and nucleated red blood cells. Cell type proportions in blood spots on Guthrie cards were estimated using a reference panel from Houseman et al. [52], and the cells included B cells, CD4 + T cells, CD8 + T cells, eosinophils, monocytes, NK cells, and neutrophils. To examine the impact of cell types on analytical findings, cell type-unadjusted DNAm was included in the analyses, and findings were further assessed using cell type-adjusted DNAm to examine any substantial changes due to cell heterogeneity. Cell type-adjusted DNAm was calculated by first regressing M-values on cell type proportions in each blood source and then treating the residuals as cell type-adjusted DNAm.

### 4.6. Descriptive Analyses

Demographic information and clinical outcomes of the complete cohort and subcohort (i.e., subjects included in the current study) were summarized and are presented as percentages. Descriptive statistics for pollen exposure in pregnancy in the subcohort were summarized and are expressed as mean ± standard deviation (SD). To compare between the complete cohort and subcohort, proportion tests were conducted for binary variables, and a chi-square test was conducted for discrete variables with more than two categories or levels, such as SES. A one-sample *t*-test was performed for pollen exposure in subjects with DNAm available to examine the agreement of the subcohort with the complete cohort.

### 4.7. Screening of CpG Sites

Our study focused on the role of DNAm in the association of pollen grain exposure during pregnancy with childhood asthma. In particular, we aimed to examine whether CpGs with at-birth DNAm, associated with PPG exposure (first arm), were further associated with childhood asthma and/or allergic rhinitis (second arm).

To avoid significant power loss, we first screened the genome-scale CpG sites, in total 294,265 sites, to detect potentially informative CpGs such that DNAm (in logit scale or M values) at birth was associated with PPG exposure. The R package ttScreening 0.0.4 was applied for this purpose with package-suggested parameter setting. The method built into ttScreening utilizes robust linear regressions and training (2/3 of the whole data) and testing (the remaining data) data to screen potentially informative sites [53]. This stage was for the purpose of screening only, and no covariates were included. For CpGs potentially associated with PPG exposure, genetic information such as mapped genes and their locations were extracted using the Illumina manifest file. The functionality of these genes was derived from NCBI, GeneCards, and UniProtKB/Swiss-Prot databases.

### 4.8. Association of Prenatal Pollen Grain Exposure and DNAm at Birth (First Arm)

A CpG site that passed screening was further examined in a full analytical model, a linear regression with DNAm at a CpG site as the dependent variable, and PPG exposure along with potential confounders as the independent variables. Maternal smoking, maternal asthma or wheeze, maternal rhinitis, socioeconomic status (SES), and sex have been shown to be associated with DNAm and were included in the model as confounders [54,55]. In our study, SES was defined based on parental occupation, number of children in a bedroom, and family income and was divided into five levels (1 to 5); the higher the level, the higher the SES status [56]. CpGs with DNAm showing statistically significant associations with pollen grain exposure during pregnancy were tested in the second arm of the analyses to assess the association of PPG exposure-related DNAm at birth with childhood allergic diseases, asthma, and allergic rhinitis.

For CpGs with DNAm associated with PPG exposure, we further examined the impact of cell type compositions by integrating cell type-adjusted DNAm into the same model. We then assessed changes in the direction of associations between PPG exposure and cell type-adjusted DNAm compared to those when cell types were not adjusted in DNAm. Since the cell types in cord blood and Guthrie cards were different, this sensitivity analysis was stratified between these two blood sources.

### 4.9. Association of Prenatal Pollen Grain Exposure-Related DNAm at Birth and Childhood Allergic Diseases (Second Arm)

The second arm of the study was to examine, among the CpGs shown to be associated with PPG exposure, the association of DNAm at birth with childhood allergic diseases, asthma, and allergic rhinitis. Logistic regressions using the SAS procedure PROC LOGISTIC were applied with childhood asthma status at age 6 years as the dependent variable (no asthma was the reference group), and DNAm at birth was the independent variable along with confounders, including maternal asthma or wheeze status, SES, passive smoke exposure during the first year of life, and sex of offspring.

For childhood allergic rhinitis status, repeated measures at ages 2, 3, and 6 years were included in the analyses using generalized linear regressions with repeated measures. In this model, DNAm and confounders were the independent variable, and status of allergic rhinitis at each age was the dependent variable (no allergic rhinitis was the reference group). The same set of confounders was included as those for childhood asthma, except that maternal rhinitis status instead of maternal asthma or wheezing status was included in the model. The SAS procedure PROC GENMOD was utilized to fit the model. For CpGs associated with asthma or allergic rhinitis, as done in the analyses of PPG exposure and DNAm at birth, we also evaluated the impact of cell type proportions on the association of DNAm with asthma and allergic rhinitis using the same approach.

**Figure 2:**
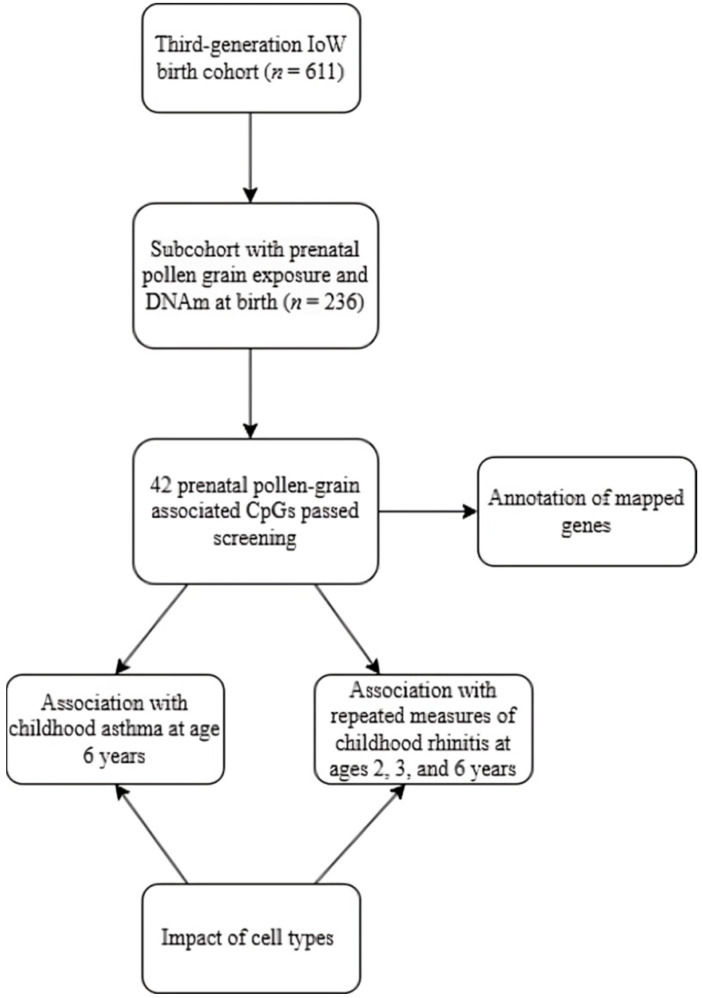
Analytic process of epigenome-wide study on pollen exposure and childhood asthma and allergic rhinitis. For the association between prenatal pollen exposure and DNAm at birth, covariates included maternal smoking, maternal asthma or wheeze, maternal rhinitis, SES, and sex of offspring. For the association between DNAm at birth and childhood allergic diseases (asthma and rhinitis), covariates included maternal asthma or wheeze, maternal rhinitis, SES, passive smoke exposure in the first year of life, and sex of offspring.

The flow of the study plan is summarized in Figure 2. In all the analyses after screening in SAS version 9 (SAS Institute, Cary, NC, USA), statistical significance was set at 0.05, and multiple testing was not adjusted since the CpGs were post-screened with potential association with prenatal exposure to pollen grains. Regression coefficients or odds ratios (OR) were estimated for each independent variable, and 95% confidence intervals (CI) were calculated.

## 5. Conclusions

In conclusion, we show that DNAm in newborns has an important role on the connection between pollen exposure in pregnancy and childhood allergic diseases. We discovered two PPG-associated CpG sites associated with asthma and one PPG-associated CpG site associated with allergic rhinitis. These findings could help in understanding the molecular pathways and processes of asthma and allergic rhinitis, potentially leading to improved prognosis of such diseases.

## Figures and Tables

**Table 1:** Comparison of complete cohort (*n* = 611) and subcohort of study subjects (*n* = 236) on potential risk factors and childhood asthma and allergic rhinitis status.

Variables * (N)	Complete Cohort *n* (*n*/N; %)	Subcohort *n* (%)	*p*-Value **
Maternal smoking (Yes)	178 (178/560; 31.79)	75 (32.75)	0.7548
Maternal asthma or wheeze (Yes)	79 (79/490; 16.12)	33 (16.02)	0.9687
Maternal rhinitis (Yes)	217 (217/492; 44.11)	97 (42.73)	0.6757
SES 1 (lowest) 2 3 4 5 (highest)	77 (77/400; 19.25) 97 (97/400; 24.25) 114 (114/400; 28.50) 61 (61/400; 15.25) 51 (51/400; 12.75)	30 (12.93) 67 (28.88) 72 (31.03) 40 (17.24) 23 (9.91)	0.0507
Sex (Male)	336 (336/600; 56.00)	122 (51.69)	0.1827
Passive smoke in the first year (Yes)	58 (58/354; 16.38)	24 (14.37)	0.4830
Childhood asthma status at age 6 (Yes)	44 (44/611; 7.20)	32 (13.56)	0.0002
Childhood allergic rhinitis at age 2 (Yes)	42 (42/287; 14.63)	24 (15.79)	0.6859
age 3 (Yes)	40 (40/271; 14.76)	24 (14.72)	0.9896
age 6 (Yes)	38 (38/181; 20.99)	24 (19.51)	0.6873

Note: * Except for SES, all other variables have two levels. Only counts and % for one category are included. The counts and % for the other categories can be readily inferred based on *n* and N values. ** *p*-values are for comparisons between the complete cohort and subcohort.

**Table 2:** List of top 10 CpGs from screening showing association with PPG exposure in terms of statistical significance.

CpG	Estimate/*p*-Value *	Gene	Gene Location	Biological Functions **
cg01375976	0.0327 (0.0001)	*ZNF3*	TSS1500	Negative regulation of transcription Nucleic acid binding Identical protein binding
cg13814708	0.0283 (0.0001)	*CAND2*	TSS200	TBP-class protein binding activity SCF complex assembly Positive regulation of DNA-templated transcription Protein ubiquitination
cg22654601	−0.0154 (0.0001)	*ATP6V1H*	TSS200; TSS1500	Mediates acidification of intracellular organelles
cg15150215	−0.0225 (0.001)	*TBL3*	TSS1500	RNA binding snoRNA binding
cg07046197	−0.0252 (0.0002)	*CDC27*	TSS1500	Controls progression through mitosis Protein phosphatase binding
cg10571824	0.0391 (0.0002)	*MAD1L1*	Body	Cell cycle control and tumor suppression
cg26202797	0.0272 (0.0002)	--	--	--
cg26525457	0.0388 (0.0002)	*CYTH1*	Body	Lipid binding Guanyl-nucleotide exchange factor activity Membrane trafficking
cg19056515	0.0297 (0.0002)	*#LOC404266*; *HOXB5*	Body; TSS200	DNA-binding transcription factor activity DNA-binding transcription activator activity
cg25644556	−0.0525 (0.0003)	*##FAM38B*	TSS1500	Monoatomic cation channel activity Mechanosensitive monoatomic cation channel activity

Abbreviations: Gene Location: TSS = transcription start site; 5′UTR = 5′ untranslated region; Biological Functions: RNA = ribonucleic acid; snoRNA = small nucleolar ribonucleic acid; SCF = Skp, Cullin, F-box; TBP = TATA-binding protein. * Estimate: Regression coefficients; *p*-value is for the estimated regression coefficient. ** Biological functions of protein coding genes were sourced from NCBI, GeneCards, and UniProtKB/Swiss-Prot gene summaries. -- CpGs with missing gene names were unavailable in the Illumina manifest file. #*LOC40426* in vivo function is unknown. ##*FAM38B* is also known as *PIEZO2*.

## Data Availability

The data presented in this study are available on request from the corresponding author.

## Supplementary Materials

The following supporting information can be downloaded at https://www.mdpi.com/article/10.3390/epigenomes9010009/s1. Table S1A: List of PPG-associated CpGs obtained from and their corresponding genetic information; Table S1B: Estimates of 41 PPG-associated CpGs for asthma direction; Table S1C: Estimates of 42 PPG-associated CpGs for allergic rhinitis direction; Table S2A: Associations of 41 PPG-associated CpGs with childhood asthma at age 6 years; Table S2B: Associations of 41 PPG-associated CpGs with repeated measures of childhood allergic rhinitis at ages 2, 3, and 6 years.

## Abbreviations

The following abbreviations are used in this manuscript:

CI	confidence interval
CpG	cytosine-phosphate-guanine
DNAm	DNA methylation
IoW	Isle of Wight
IOWTBC	The Isle of Wight third-generation birth cohort
ISAAC	International Study of Asthma and Allergy in Childhood
NK	natural killer
NRES	National Research Ethics Service
OR	odds ratio
PFAS	pollen–food allergy syndrome
PPG	prenatal pollen grain
SES	socioeconomic status
